# A Cross-Tissue Transcriptome-Wide Association Study Reveals Novel Susceptibility Genes for Diabetic Kidney Disease in the FinnGen Cohort

**DOI:** 10.3390/biomedicines13051231

**Published:** 2025-05-19

**Authors:** Menghan Liu, Zehua Li, Yao Lu, Pingping Sun, Ying Chen, Li Yang

**Affiliations:** 1 Renal Division, Peking University Institute of Nephrology, Peking University First Hospital, Beijing 100034, China; 2 Key Laboratory of Renal Disease-Ministry of Health of China, Key Laboratory of Chronic Kidney Disease Prevention and Treatment (Peking University)-Ministry of Education of China, Peking University First Hospital, Beijing 100034, China; 3 Research Units of Diagnosis and Treatment of Immune-Mediated Kidney Diseases, Chinese Academy of Medical Sciences, Peking University First Hospital, Beijing 100034, China; 4 Department of Internal Medicine, Beijing Chao-Yang Hospital, Capital Medical University, Beijing 100054, China

**Keywords:** diabetic kidney disease, cross-tissue transcriptome-wide association study, novel susceptibility genes, druggable target, Mendelian randomization

## Abstract

**Background/Objectives**: Diabetic kidney disease (DKD) is a common diabetic complication, driven by a multifactorial pathogenesis that includes various genetic components. However, the precise causative genes and their underlying biological pathways remain poorly understood. **Methods**: We performed a cross-tissue transcriptome-wide association study (TWAS) of DKD using expression quantitative trait loci (eQTL) data from 49 tissues in the Genotype—Tissue Expression (GTEx) version 8 (v8) resource. Five complementary analytical frameworks—sparse canonical correlation analysis (sCCA), functional summary-based imputation (FUSION), fine-mapping of causal gene sets (FOCUS), summary-data-based Mendelian randomization (SMR), and multi-marker analysis of genomic annotation (MAGMA)—were integrated to nominate candidate genes. Causal inference was refined using Mendelian randomization (MR), and biological significance was evaluated through pathway enrichment, protein interaction networks, and druggability profiling. **Results**: We identified 23 candidate genes associated with DKD risk, of which 13 were supported by MR analysis. Among these, 10 represent previously unreported susceptibility genes. Notably, four genes—*HLA-DRB1*, *HLA-DRB5*, *NOTCH4*, and *CYP21A2*—encode potentially druggable proteins, with *HLA-DRB5* and *CYP21A2* both qualifying as novel susceptibility genes and therapeutic targets. These genes converge on immune modulation, steroid biosynthesis, DNA repair, and transcriptional regulation—processes central to DKD pathogenesis. **Conclusions**: Our study represents the first systematic cross-tissue TWAS of DKD, revealing a prioritized set of genetically and functionally supported susceptibility genes. The identification of druggable targets among these genes provides critical insight into the mechanistic underpinnings of DKD and highlights their potential for future therapeutic development. These findings enhance our understanding of DKD pathophysiology and offer a foundation for precision medicine strategies in nephrology.

## 1. Introduction

Diabetic kidney disease (DKD) is a leading microvascular complication of diabetes and ranks among the principal drivers of chronic kidney disease (CKD) and end-stage renal disease (ESRD) worldwide. Current estimates suggest that DKD accounts for 30–50% of all CKD cases globally, with its prevalence rising in parallel with the diabetes pandemic [1,2]. In Europe, DKD affects approximately 25–47% of individuals with diabetes [3], underscoring its substantial burden. DKD frequently progresses to ESRD, necessitating renal replacement therapy. National data from Germany indicate that approximately 61% of patients beginning chronic renal replacement therapy have diabetes [4]. Clinically, DKD remains difficult to manage with standard interventions, including intensive glycemic and blood pressure control and renin–angiotensin system blockade, which often merely delay and rarely prevent renal decline [5]. Moreover, DKD markedly increases the risk of macrovascular and microvascular complications, such as cardiovascular disease, peripheral arterial disease, and refractory vascular ulcers, further worsening clinical outcomes [6]. These epidemiological and clinical observations highlight the urgent need to elucidate the underlying genetic mechanisms of DKD and identify its key susceptibility genes. To date, GWAS has identified 41 risk loci significantly associated with DKD [7]. However, despite the power of GWAS in discovering genetic loci, 90% of identified variants reside in non-coding regions, and complex linkage disequilibrium (LD) patterns can obscure the identification of causal variants [8]. Additionally, regulatory variants that account for substantial heritability are often not well-captured by GWAS [9]. DKD progression is influenced by multiple genetic variants, each with distinct heritable effects, highlighting its polygenic susceptibility [7]. These challenges limit the statistical power and biological interpretability of GWAS, complicating the accurate identification of susceptibility genes [10].

Transcriptome-wide association study (TWAS) offers a novel approach for identifying disease risk genes and causal genes at loci identified by GWAS through the systematic integration of expression quantitative trait loci (eQTL) with GWAS statistics [11]. As an extension of GWAS methodology, TWAS enhances statistical power, reduces false positives, and improves biological interpretation and clinical translation [11]. Several TWAS algorithms have been developed, including functional summary-based imputation (FUSION), fine-mapping of causal gene sets (FOCUS), MetaXcan, and summary-data-based mendelian randomization (SMR) [11,12,13,14]. Recent advancements in the cross-tissue TWAS framework leverage tissue-specific transcriptional regulation and shared regulatory landscapes across different tissues, thereby improving gene imputation accuracy. Approaches such as unified test for molecular signatures (UTMOST) and sparse canonical correlation analysis (sCCA) have proved to be effective in elucidating complex causal gene associations [9,15]. By combining cross-tissue and single-tissue TWAS, researchers can validate findings and identify key genes with pleiotropic effects across tissues, providing comprehensive insights into disease pathogenesis. Recently, these integrative TWAS strategies have been used to identify novel susceptibility genes for conditions such as migraine [16], coronary atherosclerosis [17], ulcerative colitis [18], and CKD [19].

Currently, there is a notable absence of cross-tissue TWAS research in DKD, especially concerning multi-organ mechanisms, such as the brain–kidney, heart–kidney, and adipose–kidney axes, which are increasingly recognized as important contributors to DKD pathogenesis. For example, the renin–angiotensin system (RAS) signaling between the brain and kidney has been implicated in neural regulation of renal function in diabetic states [20]. Cardiorenal interactions, including myocardial injury-induced renal impairment, have also been shown to be exacerbated in diabetes [21]. Moreover, lipid dysregulation through VEGF-B-mediated fatty acid transport in the adipose–kidney axis contributes to renal lipotoxicity and tubular damage [22]. These axes exemplify the importance of inter-organ crosstalk in shaping the molecular landscape of DKD [23]. In this study, we employed five distinct cross-tissue and single-tissue TWAS methods to systematically integrate DKD GWAS data with Genotype–Tissue Expression (GTEx) version 8 (v8) eQTL files to screen for potential pathogenic genes associated with DKD. We identified twenty-three novel susceptibility genes and further validated thirteen of these genes through Mendelian randomization (MR) analysis to pinpoint the true causal genes for DKD. Finally, we assessed the proteins encoded by the MR-validated genes for their potential as drug targets. Our primary objective was to systematically identify and validate susceptibility genes causally associated with DKD. The secondary objective was to evaluate the druggability of the validated genes and explore their potential as therapeutic targets for DKD.

## 2. Materials and Methods

### 2.1. Study Design

**Figure 1:**
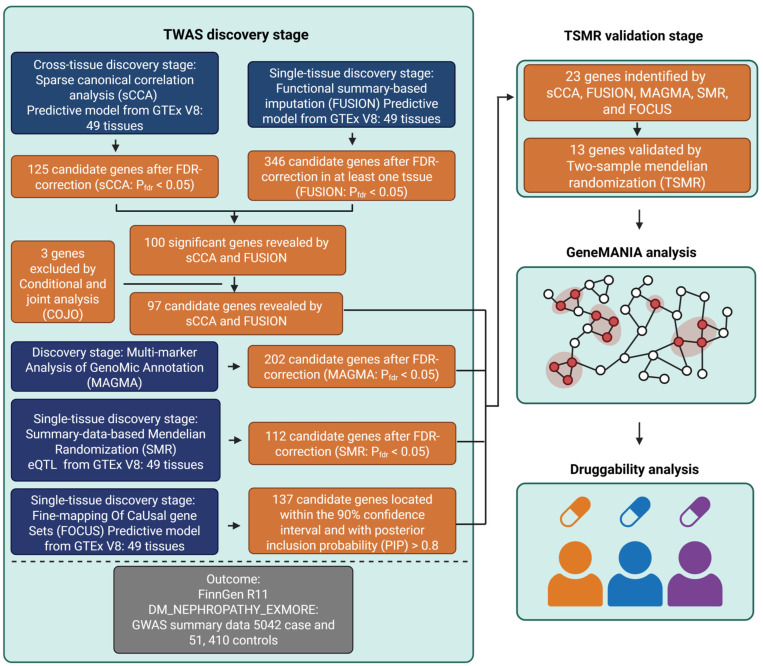
Systematic workflow of multi-stage exploratory TWAS analysis in DKD. Schematic representation of the analytical pipeline integrating multiple TWAS approaches. The workflow begins with cross-tissue discovery using sCCA on GTEx V8 data (49 tissues) and FinnGen R11 GWAS data (5042 cases, 51,410 controls). Five parallel single-tissue discovery methods (SMR, MAGMA, FUSION, sCCA, and FOCUS) were applied, identifying different sets of candidate genes. In the validation phase, MR analysis was utilized and yielded 13 candidate genes for DKD. Functional enrichment analysis and protein interaction network analysis were employed to elucidate the functions of these positive susceptibility genes. Ultimately, we conducted druggability analysis to reveal the potential of these genes as therapeutic targets. Figure 1 created with BioRender.com, with permission.

The overall study design is summarized in Figure 1. In brief, we employed a multi-stage exploratory strategy to identify susceptibility genes for DKD. First, DKD GWAS summary statistics from the FinnGen R11 cohort (5042 cases and 51,410 controls) and eQTL data from 49 human tissues in the GTEx v8 dataset were selected as outcome and exposure data, respectively. A cross-tissue TWAS analysis was initially performed using sCCA to capture shared genetic effects across multiple tissues. In parallel, we conducted a single-tissue discovery stage using multiple complementary approaches, including FUSION-based TWAS, multi-marker analysis of genomic annotation (MAGMA) gene-level association analysis, SMR for causal inference, and FOCUS for fine-mapping. Conditional and joint (COJO) analysis was applied to eliminate false positives caused by LD. Genes supported by multiple lines of evidence were further evaluated in a validation stage using two-sample Mendelian randomization (TSMR) to confirm causality. We then carried out functional network analyses using the GeneMANIA, GO, KEGG, and Reactome pathways to explore their biological relationships and used druggability analysis to assess their therapeutic potential.

### 2.2. DKD GWAS Data Source

GWAS summary statistics for DKD were obtained from the FinnGen R11 public release (https://www.finngen.fi/en, accessed on 8 October 2024). DKD cases were defined by the DM_NEPHROPATHY_EXMORE endpoint, using International Classification of Diseases (ICD-10) codes; full definitions are available in the FinnGen Risteys phenotype browser (https://risteys.finngen.fi/endpoints/DM_NEPHROPATHY_EXMORE, accessed on 8 May 2025).

### 2.3. eQTL Files Source

The eQTL data were sourced from the GTEx project v8 database, which offers extensive gene expression profiles across 49 human tissues obtained from 838 post-mortem donors. This dataset is publicly available via the European Bioinformatics Institute repository (https://ftp.ebi.ac.uk/pub/databases/spot/eQTL/imported/GTEx_V8, accessed on 8 October 2024).

### 2.4. Cross-Tissue TWAS Analysis Using sCCA

We performed cross-tissue TWAS using sCCA based on GTEx v8 multi-tissue eQTL panels. sCCA integrates gene expression across tissues by learning a sparse linear combination of tissue-specific expression levels optimized to maximize correlation with cis-genetic variants. These canonical loadings reflect each tissue’s contribution to the predictor and allow for feature selection while maximizing genotype–expression correlation, thus offering advantages over weighted averages or PCA approaches [15]. We tested sCCA-derived cross-tissue predicted expression against DKD GWAS data and integrated these results with single-tissue TWAS using the aggregate Cauchy association test (ACAT). All analyses used autosomal chromosomes, default parameters, and False Discovery Rate (FDR)-adjusted significance threshold (*p* < 0.05). LD information was derived from the 1000 Genomes Project (Phase 3) European reference panel.

### 2.5. Single-Tissue TWAS Analysis Using FUSION

We employed the FUSION toolkits (http://gusevlab.org/projects/fusion/, accessed on 10 October 2024) to conduct a TWAS analysis that integrates DKD GWAS data with eQTL data from GTEx V8 across 49 tissues, aiming to evaluate the association of each gene with the disease [11]. Initially, we estimated the LD between the predictive model and the single-nucleotide polymorphisms (SNPs) at each GWAS locus using samples from the 1000 Genomes Project representing individuals of European ancestry. Subsequently, FUSION combines multiple predictive models, namely, best linear unbiased prediction (BLUP), Bayesian Sparse Linear Mixed Model (BSLMM), Least Absolute Shrinkage and Selection Operator (LASSO), Elastic Net, and top 1, to evaluate the cumulative impact of SNPs on gene expression weights. The model with the highest predictive accuracy was then chosen to determine the gene weights [24]. Following this, we integrated the genetic effect of DKD, represented by the DKD GWAS Z-score, with these gene weights to perform the TWAS analysis for DKD. Genes with an FDR of <0.05 in at least one tissue were considered significant in the FUSION analysis.

### 2.6. Conditional and Joint Analysis

To identify independent genetic signals within the identified loci, we conducted COJO analyses using the FUSION post-processing module [11]. This approach considered the LD patterns between markers, thereby enhancing the identification of truly independent genetic associations [25]. Genes that yielded positive results in both sCCA and FUSION analyses were included in the COJO analysis. Signals that retained significance after the COJO analysis were classified as jointly significant, while those that weakened were categorized as marginally significant. Genes lacking joint significance were excluded from further analysis.

### 2.7. Gene-Based Association Analysis

Gene-based association analyses were performed using MAGMA software (v1.08). This analytical framework systematically integrated SNP-level summary statistics to generate comprehensive gene-wise association metrics by evaluating the cumulative impact of multiple SNPs assigned to a designated gene (±10 kb). The analysis adhered to standard MAGMA protocols with default parameters, as outlined in the software’s technical documentation [26]. The reference panel for the MAGMA analysis was sourced from phase 3 of the 1000 Genomes Project, concentrating on the European population to compute the LD.

### 2.8. Single-Tissue TWAS Analysis Using Batch SMR

We further employed the SMR method to filter and enhance the results obtained from other TWAS methods. The analysis was conducted using the SMR analysis program available on the SMR website (https://yanglab.westlake.edu.cn/software/smr, accessed on 10 October 2024) [27]. We utilized the SMR method to perform linear regression on DKD GWAS data and eQTL data from GTEx V8 across 49 tissues. Additionally, we applied the HEIDI method to systematically test for heterogeneity in instrumental SNP associations, thereby distinguishing true linkage from horizontal pleiotropy. Instrumental SNPs with significant heterogeneity (unadjusted HEIDI *p*-value ≤ 0.01) were excluded to ensure the robustness of SMR-based gene prioritization. Genes with an FDR of <0.05 in at least one tissue were considered significant in the batch SMR analysis.

### 2.9. Single-Tissue TWAS Analysis Using FOCUS

We employed the FOCUS (pyfocus version 0.802) software to conduct fine-mapping of TWAS related to genomic risk regions. This tool integrates GWAS data and eQTL weights as inputs, generating a set of credible genes as outputs to aid in elucidating the identified genomic risk [12]. The FOCUS database used in our analysis was derived from gene expression prediction weight data from GTEx V8 across 49 tissues. In our analysis, we identified DKD risk genes by applying the following criteria: (1) a posterior inclusion probability (PIP) threshold of >0.8; (2) a significance level of *p* < 5 × 10^−8^; and (3) genes included within the 90% confidence intervals (CIs).

### 2.10. Two-Sample MR

We conducted MR analysis using the TwoSampleMR (version 0.6.6) package to examine causal relationships. Cis-eQTL SNPs were utilized as instrumental variables (IVs), with gene expression considered as exposure and DKD as the outcome. We first identified genome-wide significant SNPs (*p*-value < 5 × 10^−8^) and performed LD clumping to select independent SNPs (r^2^ < 0.001) [28]. Given that only one independent genetic instrument was available per gene, MR effect estimates were calculated using the Wald ratio method, with a significance threshold of *p*-value < 0.05.

### 2.11. Over-Representation Analysis

In the context of functional enrichment analysis, we performed Gene Ontology (GO), Kyoto Encyclopedia of Genes and Genomes (KEGG), and Reactome pathway analyses using over-representation analysis (ORA) via the ‘clusterProfiler’ package (version: 4.16.0) for GO and KEGG annotations, and the ‘ReactomePA’ package (version: 1.52.0) for Reactome pathway analysis [29]. The 13 positive genes identified in the MR analysis served as the input gene list. Enrichment scores were calculated using hypergeometric tests, with a significance threshold set at *p* < 0.05 to determine enriched terms. Graphical representations of the enriched pathways were generated using ‘enrichplot’ to enhance the interpretation of biological significance through network plots.

### 2.12. GeneMANIA Analysis

The functional interpretation of the target genes was conducted using GeneMANIA (http://genemania.org, accessed on 24 December 2024), a comprehensive platform for gene function prediction. This analytical tool integrates various biological networks, including genetic interactions, molecular pathways, and co-expression patterns, to elucidate the functional relationships and biological processes associated with our gene set of interest.

### 2.13. Druggability Assessment and Tissue-Specific Expression Analysis

To assess whether the MR-validated genes can function as druggable targets, the genes that passed the primary analysis were compared with 4479 genes identified as part of the drugged or druggable genome by Finan et al. [30]. Three tiers were established based on the target’s position within the drug development pipeline. Tier 1 (1427 genes) encompasses efficacy targets for approved small molecules and biotherapeutic agents, as well as drug candidates currently in clinical phases. Tier 2 (682 genes) includes encoding targets known to have bioactive drug-like small molecule binding partners, as well as those exhibiting ≥ 50% sequence identity with approved drug targets. Tier 3 (2370 genes) comprises encoding targets that share more distant similarities with approved drug targets, along with members of key druggable gene families that are not classified within Tiers 1 or 2 [30]. Furthermore, we annotated the drug target development phases and the associated drugs using the Therapeutic Target Database (http://db.idrblab.net/ttd/, accessed on 8 May 2025). For identified druggable targets, we evaluated their tissue-specific expression patterns to assess their therapeutic relevance to DKD. We utilized publicly available data from The Human Protein Atlas (HPA, https://www.proteinatlas.org/, accessed on 8 May 2025) for protein expression and the GTEx project database for RNA expression. For each gene of interest, we directly retrieved standardized expression visualizations from the HPA portal, focusing on tissues relevant to DKD pathophysiology, including the kidney, adrenal gland, immune system components, and vascular tissues. The HPA database provided protein expression data based on immunohistochemistry categorized as high, medium, low, or not detected, while the GTEx dataset offered RNA expression profiles quantified as normalized transcripts per million (nTPM). These tissue expression profiles were compiled to provide a comprehensive view of the expression patterns of potential druggable targets and to evaluate their biological plausibility in DKD-relevant tissues.

### 2.14. Ethic Approval

This study only used publicly available data, and, hence, no ethics approval was required. Details of ethical approval and participant consent for each of the studies that contributed to the GWAS can be found in the original publications.

## 3. Results

### 3.1. Discovery of DKD Causal Genes Through Integrative TWAS Analysis

**Figure 2:**
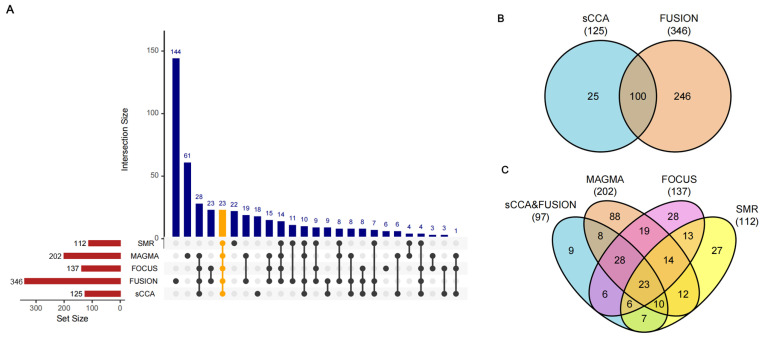
Multi-method integration of TWAS approaches in DKD. Intersection plot (**A**) and Venn plots (**B**,**C**) showing overlapping genes identified by different analytical methods. In the intersection plot (**A**), the upper panel displays intersection sizes, with bar height indicating the number of overlapping genes. The lower panel shows the total gene sets identified by each method (red bars). Black dots and connecting lines indicate specific method combinations. A total of 346, 202, 137, 125, and 112 genes were identified by FUSION, MAGMA, FOCUS, sCCA, and SMR, respectively. Twenty-three genes were consistently detected by all five methods, which are highlighted in orange.

We conducted a TWAS analysis that integrated five different approaches, utilizing exposure data from GTEx V8 across 49 tissues and outcome data derived from the DKD GWAS summary statistics of FinnGen R11, which included 5042 cases and 51,410 controls (Figure 1). Initially, we employed sCCA for the cross-tissue discovery phase, which identified 125 candidate genes after FDR correction (Supplementary Tables S1 and S2). Subsequently, single-tissue FUSION analysis yielded 346 significant genes with an FDR < 0.05 in at least one tissue (Supplementary Figure S1, Supplementary Table S3). The integration of FUSION results with those from sCCA revealed 100 overlapping significant genes (Supplementary Table S4). To reduce the likelihood of false positives due to LD, we conducted COJO analysis, which led to the exclusion of three genes—*ZNF184*, *HSPA1L*, and *PPT2* (Supplementary Table S5). The remaining 97 genes were further filtered through multiple complementary TWAS approaches (Figure 1). In total, we identified 202 and 112 positive genes with FDR < 0.05 in the MAGMA and batch SMR analysis, respectively (Supplementary Tables S6 and S7). By applying a strict threshold of posterior inclusion probabilities (PIP) > 0.8 and a 90% confidence interval level, the FOCUS analysis identified 137 candidate genes through fine-mapping (Supplementary Table S8). We calculated the mean and median values of PIP, transcriptome-wide association Z-score, and FDR for FOCUS-positive genes across multiple tissues (overall mean/median: PIP = 0.966/0.999, Z-score = 2.19/0.880, FDR = 0.00676/4.38 × 10^−12^) (Supplementary Table S9). The intersection analysis, as illustrated in the upset plot, revealed varying degrees of overlap among these methods (Figure 2A). Notably, 23 genes were shared by all five methods, which include major histocompatibility complex (MHC) genes (*HLA-DQA1*, *HLA-DQB1*, *HLA-DRB1*, *HLA-DRB5*, *HLA-DMA*, and *HLA-DMB*), alongside 17 non-MHC genes (*PHTF1*, *ZSCAN9*, *ZNF165*, *ZKSCAN8*, *NOTCH4*, *AGER*, *PRRT1*, *SKIV2L*, *NELFE*, *VWA7*, *MSH5*, *APOM*, *MICB*, *POU5F1*, *CLIC1*, *DDAH2*, and *CYP21A2*) (Figure 2B,C).

### 3.2. Validation of DKD Susceptibility Genes Through Tissue-Specific MR

**Figure 3:**
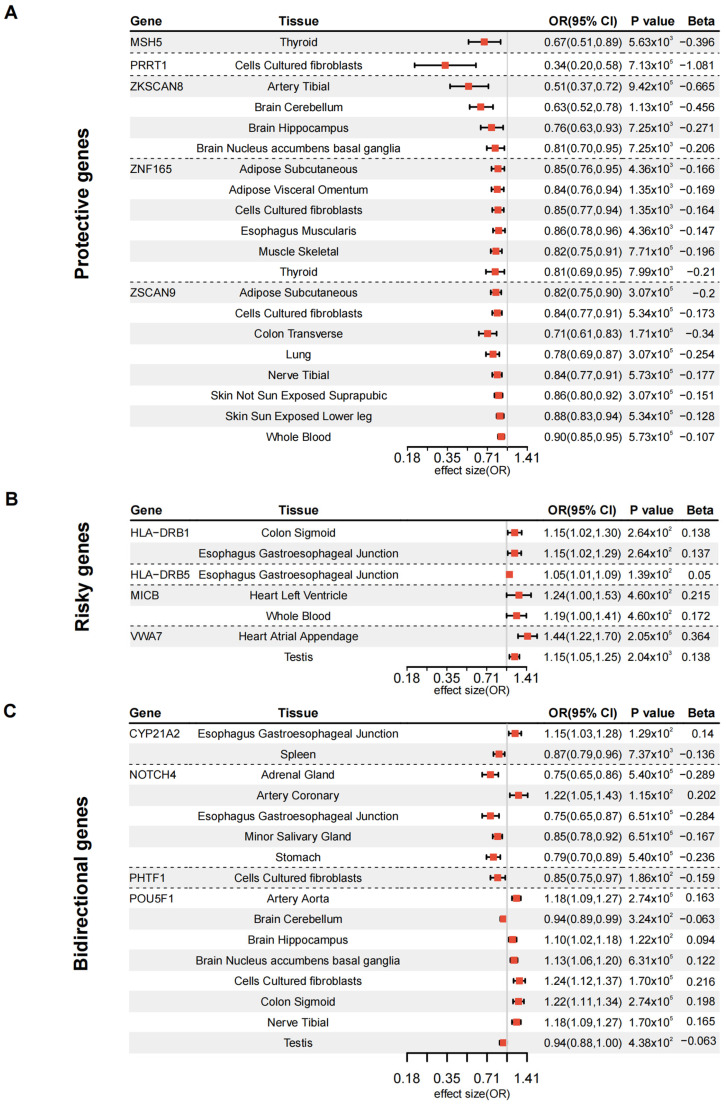
Tissue-specific genetic effects of DKD-associated genes. Forest plots showing odds ratios (OR) and 95% confidence intervals for gene-tissue pairs. Protective genes (**A**), risk genes (**B**), and bidirectional genes (**C**) were presented separately. Effect sizes are displayed on the log scale, with OR > 1 indicating risk-increasing and OR < 1 indicating protective effects. Significant associations (*p* < 0.05) are shown for 13 genes across multiple tissues.

To validate the causal relationships of these 23 genes with DKD, we performed MR analysis. A total of 13 genes were identified, showing significant associations with DKD in specific tissues, as illustrated in the forest plot (Figure 3, Supplementary Table S10). These genes can be classified into three groups based on their functional concordance across various tissues. The first group, comprising six genes, exhibited a consistent protective effect on DKD across all tissues (Figure 3A). These include *MSH5* (OR = 0.67, 95% CI: 0.51–0.89, *p* = 5.63 × 10^−3^), *PHTF1* (OR = 0.85, 95% CI: 0.75–0.97, *p* = 1.86 × 10^−2^), *PRRT1* (OR = 0.34, 95% CI: 0.20–0.58, *p* = 7.13 × 10^−5^), *ZKSCAN8* (OR ranging from 0.51 to 0.81, *p* values from 9.42 × 10^−5^ to 7.99 × 10^−3^), *ZNF165* (OR ranging from 0.81 to 0.86, *p* values from 4.36 × 10^−3^ to 1.35 × 10^−3^), and *ZSCAN9* (OR ranging from 0.71 to 0.90, *p* values from 5.34 × 10^−5^ to 3.07 × 10^−5^). The second group of genes exhibited a consistent risk association across different tissues (Figure 3B), including *HLA–DRB1* (OR = 1.15, 95% CI: 1.02–1.30, *p* = 2.64 × 10^−2^), *HLA–DRB5* (OR = 1.05, 95% CI: 1.01–1.09, *p* = 1.39 × 10^−2^), *MICB* (OR ranging from 1.19 to 1.24, *p* = 4.60 × 10^−2^), and *VWA7* (OR ranging from 1.15 to 1.44, *p* values from 2.04 × 10^−3^ to 2.05 × 10^−5^). The final group of three genes showed bidirectional associations across tissues (Figure 3C). Specifically, *CYP21A2* showed a positive association in the esophagus–gastroesophageal junction (OR = 1.15, 95% CI: 1.03–1.28, *p* = 1.29 × 10^−2^), but an inverse association in the spleen (OR = 0.87, 95% CI: 0.79–0.96, *p* = 7.37 × 10^−3^). *NOTCH4* demonstrated a directionally positive association in the artery coronary (OR = 1.22, 95% CI: 1.05–1.43, *p* = 1.15 × 10^−2^), whereas inverse associations were observed in the adrenal gland, esophagus gastroesophageal junction, minor salivary gland, and stomach (OR range: 0.75–0.85, *p*-values from 5.40 × 10^−5^ to 6.51 × 10^−5^). *POU5F1* was inversely associated with DKD in the brain cerebellum and testis (OR = 0.94, 95% CI: 0.88–1.00, *p* = 4.38 × 10^−2^ and 3.24 × 10^−2^, respectively), but showed positive associations across multiple other tissues, including the artery aorta, brain hippocampus, brain nucleus accumbens basal ganglia, cultured fibroblasts, colon sigmoid, and nerve tibial (OR ranging from 1.10 to 1.24, *p*-values from 1.22 × 10^−2^ to 1.70 × 10^−5^) (Figure 3C, Supplementary Table S10). Ten genes (*MSH5*, *PHTF1*, *PRRT1*, *ZSCAN9*, *ZNF165*, *ZKSCAN8*, *MICB*, *VWA7*, *POU5F1*, and *CYP21A2*) showed no prior association with DKD in systematic searches of PubMed, GWAS Catalog [31], Open Targets [32], and ClinVar [33] databases, representing novel susceptibility candidates.

### 3.3. Enrichment and Network Analysis Reveal Functional Gene Clusters in DKD Pathogenesis

**Figure 4:**
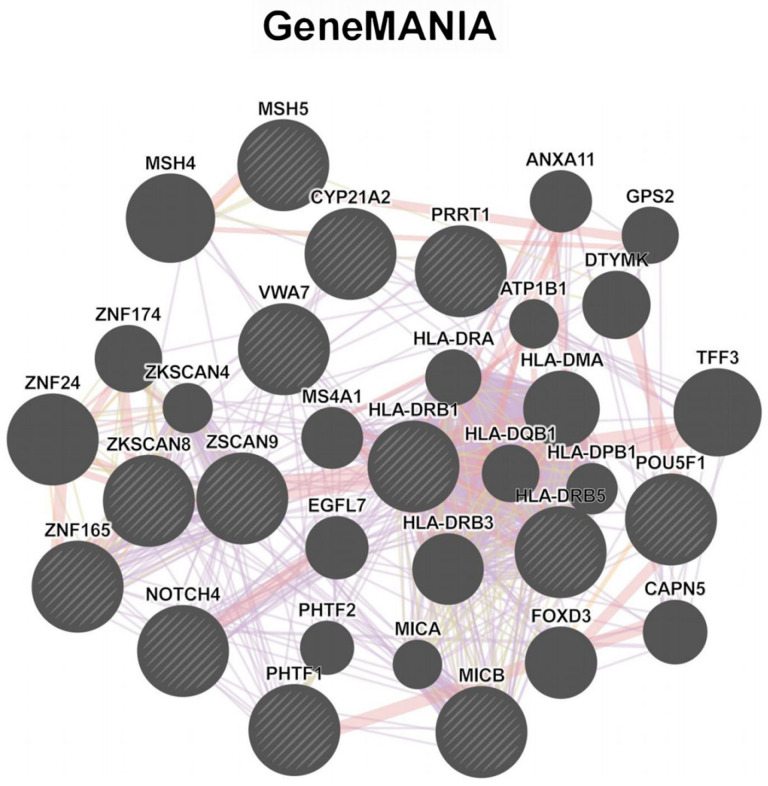
GeneMANIA gene network of DKD-associated genes. Interaction network indicated by GeneMANIA among identified genes. Node size represents gene significance in TWAS analysis (indicated by striped shading). Edges represent gene–gene interactions, with different colors indicating distinct interaction types.

To understand the functional networks of the 13 susceptibility genes associated with DKD, we performed functional enrichment analysis using GO (Supplementary Figure S2A), KEGG (Supplementary Figure S2B), and Reactome (Supplementary Figure S2C) through ORA. Additionally, we conducted network analysis using GeneMANIA (Figure 4, Supplementary Table S11). From the interaction patterns among these DKD susceptibility genes, multiple functional modules were identified. The most prominent gene cluster was orchestrated by HLA complex genes (*HLA-DRB1*, *HLA-DRB5*) and *MICB*, highlighting their roles in antigen processing, presentation, and immune response (Figure 4, Supplementary Figure S2A–C, Supplementary Table S11). *NOTCH4*, recognized as a significant participant in podocyte apoptosis [34], emerged as a key hub gene connected to both the HLA complex and zinc finger protein clusters, indicating its role in linking immune response and transcriptional regulation (Figure 4, Supplementary Figure S2A–C). The hub gene *CYP21A2* encodes a member of the cytochrome P450 superfamily of enzymes, which is essential for the synthesis of steroid hormones, including cortisol and aldosterone. Additional functional groups identified included DNA mismatch repair (*MSH5*), membrane trafficking components (*VWA7*, *PRRT1*, *PHTF1*), and transcriptional regulation and cell fate determination (*ZKSCAN8*, *ZSCAN9*, *ZNF165,* and *POU5F1*) (Figure 4, Supplementary Figure S2A–C, Supplementary Table S11).

### 3.4. MR-Validated Genes as Drug Targets

For MR-validated genes, we assessed whether the proteins they encode could serve as drug targets or be used in drug therapy. Comparing our findings with the dataset from Finan et al. [30] and the Therapeutic Target Database, we identified that 4 of the 13 genes encode proteins with druggable targets. Among these, the proteins encoded by *HLA-DRB1* and *HLA-DRB5* were classified as Tier 1 druggable targets, with Glatiramer acetate and Apolizumab currently in clinical trial development. In contrast, the proteins encoded by *CYP21A2* and *NOTCH4* were categorized as Tier 3 targets, with BBP-631, Parsatuzumab (MEGF0444A), and Crenigacestat (LY3039478) also in clinical trial development (Table 1). To further evaluate the therapeutic relevance of the four MR-validated druggable genes (*HLA-DRB1*, *HLA-DRB5*, *NOTCH4*, and *CYP21A2*), we examined their tissue-specific expression profiles using the GTEx and Human Protein Atlas datasets, with a particular focus on tissues implicated in DKD pathophysiology, such as the kidney, adrenal gland, immune system, and vasculature. *HLA-DRB1* and *HLA-DRB5* showed high expression in immune-related tissues, including lymph nodes, spleen, and bone marrow, suggesting their roles in immunopathology associated with DKD. *NOTCH4* showed high expression in the adrenal gland, and was also expressed in vascular tissues, adipose tissue, and the kidney, indicating potential involvement in endothelial signaling and metabolic regulation relevant to DKD pathophysiology. *CYP21A2* exhibited extremely high, adrenal-specific expression (nTPM >1500), consistent with its known endocrine function and possible link to adrenal–renal axis dysregulation in DKD. These expression profiles reinforce the biological plausibility and tissue-level accessibility of these targets, supporting their prioritization for DKD therapy development (Supplementary Figure S3).

## 4. Discussion

DKD is a major contributor to end-stage renal failure and cardiovascular death, representing a significant global health burden. This underscores the urgent need for a deeper mechanistic understanding and the development of targeted therapies. Cross-tissue TWAS allows deeper insights into the genetic factors underlying human DKD by systematically investigating susceptibility genes. Our study advances the methodology by integrating multiple cross-tissue and batch single-tissue TWAS strategies. This approach overcomes limitations of previous kidney disease genetic studies, which have typically relied on single-method or single-tissue analyses. Each method employed has complementary strengths: sCCA effectively identifies complex expression–phenotype correlations; FUSION enhances eQTL data integration; FOCUS improves the localization of causal variants; and SMR offers insights through MR. Furthermore, the cross-tissue sCCA-ACAT algorithm improves statistical power by capturing regulatory patterns across tissues, revealing tissue-specific effects overlooked by traditional single-tissue analyses. Consequently, our multi-method TWAS framework, which combines sCCA, FUSION, MAGMA, SMR, and FOCUS, substantially increased statistical power and enriched biological interpretation through methodological triangulation. This innovative pipeline successfully identified novel associations previously undetected in DKD genetic studies and mitigated method-specific biases, as demonstrated in recent analyses of complex traits.

Leveraging GTEx v8 eQTL data from 49 tissues, we conducted the first cross-tissue TWAS of human DKD. Among 23 genes jointly identified by all five integrative methods, 13 were validated through tissue-specific MR analysis. Of these 13, 10 showed no prior evidence of direct association with DKD based on a systematic search of PubMed, GWAS Catalog [31], Open Targets [32], and ClinVar [33], and are thus considered previously unreported susceptibility genes, including *MSH5*, *PHTF1*, *PRRT1*, *ZSCAN9*, *ZNF165*, *ZKSCAN8*, *MICB*, *VWA7*, *POU5F1*, and *CYP21A2*. The remaining three (*HLA-DRB1*, *HLA-DRB5*, and *NOTCH4*) have previously yielded evidence of association, but this was further refined and prioritized here through our analytical framework. Notably, 4 of the 13 validated genes (*HLA-DRB1*, *HLA-DRB5*, *CYP21A2*, and *NOTCH4*) encode proteins with druggable properties, and two of them (*HLA-DRB5* and *CYP21A2*) are both novel and pharmacologically actionable. Functional enrichment and network analyses revealed distinct biological roles across gene categories—risk-associated genes were linked to antigen presentation and immune regulation; some genes were protective against DNA repair, membrane trafficking, and transcriptional control; and genes with potential bidirectional associations (*NOTCH4*, *CYP21A2*, *POU5F1*) might be linked to processes such as steroid biosynthesis, angiogenesis, and cell fate determination. However, these potential associations require further investigation, as bidirectionality may reflect methodological artifacts or true context-dependent roles.

*HLA-DRB1*, *MICB*, and *NOTCH4* have been implicated in DKD through GWAS and experimental investigations. Notably, HLA-DRB1*04 is associated with protective effects, while HLA-DRB1*03 correlates with an elevated risk of microalbuminuria. *MICB* was identified as a risk gene via MR analyses of protein quantitative trait loci data. The experimental findings indicate that *NOTCH4* activation may promote DKD progression by suppressing *KLF4* and inducing podocyte apoptosis mediated by Bcl-2/p53. The replication of these DKD susceptibility genes in our analysis reinforces the reliability and reproducibility of our cross-tissue TWAS approach.

Furthermore, our TWAS research identified novel susceptibility genes for DKD, including *HLA-DRB5*, a well-characterized MHC-II antigen-presenting molecule implicated in the pathogenesis of various autoimmune diseases [35,36]. The activation of CD8+ T cells via *HLA-DRB5* is a recognized mechanism in autoimmune disorders [37]. Although meta-analyses have shown a positive relationship between *HLA-DRB5* deficiency and diabetes risk [38], there is a lack of reports linking *HLA-DRB5* to DKD pathogenesis. In our TWAS and MR analysis, *HLA-DRB5* demonstrated a mild risk effect (OR = 1.05) and was identified as a contributor to increased DKD risk. Elevated proportions of CD8+ T cells were observed in kidney tissues from both DKD patients and animal models [39], suggesting that *HLA-DRB5* may enhance DKD risk by activating T cell immune responses through antigen presentation.

Increased glucocorticoid metabolism has been documented in DKD [40], with serum cortisol levels positively correlating with microalbuminuria in diabetic patients [41,42]. Enhanced activity of the hypothalamic–pituitary–adrenal (HPA) axis may independently contribute to CKD risk in type 2 diabetes patients [43]. *CYP21A2*, which encodes 21-hydroxylase, is a crucial enzyme in the glucocorticoid synthesis pathway [44]. Our TWAS and MR analyses suggest a causal link between *CYP21A2* and the development of DKD.

An evaluation of druggability indicates that proteins encoded by *HLA-DRB1*, *HLA-DRB5*, *CYP21A2*, and *NOTCH4* hold potential applicability as drug targets in clinical trials. Apolizumab (Hu1D10, Remitogen), a humanized IgG1 monoclonal antibody targeting the HLA-DR β-chain, has shown therapeutic efficacy in patients with follicular lymphoma [45]. Glatiramer acetate, composed of four natural amino acids—L-glutamic acid, L-alanine, L-tyrosine, and L-lysine—is employed in treating relapsing forms of multiple sclerosis (MS) in adults. BBP-631, an AAV5 gene therapy bioreagent, is used for the treatment of congenital adrenal hyperplasia (CAH). Parsatuzumab (MEGF0444A), a humanized monoclonal antibody targeting *EGFL7*, exhibits immunomodulatory effects and has demonstrated anti-angiogenic activity, tumor growth inhibition, and improved survival in various xenograft and genetically engineered mouse tumor models. Crenigacestat (LY3039478) is a potent inhibitor of Notch and gamma-secretase, having completed Phase I safety evaluations primarily in advanced solid tumors and T-cell lymphoblastic leukemia (NCT02836600, NCT02518113). The DKD susceptibility genes identified through our TWAS combined with MR analysis offer promising avenues of research into drug therapy targets, facilitating the potential repurposing of existing marketed or developmental drugs.

Beyond the extensively studied genes discussed above, our investigation also uncovered several additional validated genes whose functional relevance to DKD warrants further exploration. Through TWAS and MR analysis, we identified *MSH5*, *ZNF165*, *ZKSCAN8*, and *ZSCAN9* as novel protective factors exhibiting moderate protective effects (ORs generally ranging from 0.51 to 0.85) against DKD. Notably, zinc finger proteins (ZNFs) and *MSH5* (MutS Homolog 5) play critical roles in DNA damage repair (DDR) [46,47,48], suggesting that their protective effects may be mechanistically linked to their DDR functions. *POU5F1*, which encodes OCT4, is a recognized reprogramming factor in induced pluripotent stem cells (iPSCs) and has been associated with enhanced angiogenesis [49,50]. Our results suggest a modest but significant contribution of OCT4 to the progression of DKD.

*PRRT1* encodes a transmembrane AMPAR-associated protein that modulates excitatory synaptic strength and density and exhibits a strong protective effect (OR = 0.34) against DKD. *PHTF1*, a putative homeobox gene primarily involved in transcriptional regulation, showed a moderate protective effect, although its specific molecular functions have yet to be thoroughly investigated [51]. *VWA7*, a member of the von Willebrand factor A domain protein family, has been linked to fasting plasma glucose levels in GWAS studies [52,53], although its renal-specific role remains unclear. Although the current literature is sparse for these genes, their known biological functions suggest plausible roles in DKD susceptibility, highlighting the need for future experimental validation. Collectively, these findings emphasize the diverse molecular pathways through which the full set of 13 validated genes may influence DKD pathogenesis.

Our findings provide translational insight with direct relevance to clinical practice. The genetically and transcriptionally supported susceptibility genes identified in this study may inform future risk stratification strategies for DKD, enabling the earlier identification of high-risk individuals prior to overt renal dysfunction. The integration of these loci into multi-omic prediction models could support the development of gene-based diagnostics for preclinical DKD. In parallel, the identification of druggable targets—including *HLA-DRB1*, *HLA-DRB5*, *NOTCH4*, and *CYP21A2*—underscores the potential for therapeutic innovation. Notably, *HLA-DRB5* and *CYP21A2* represent both novel susceptibility genes and pharmacologically actionable targets, highlighting their dual relevance to disease causation and intervention. These genes converge on immune modulation and steroid biosynthesis—mechanistic pathways with existing or emerging therapeutic modalities. Such insights offer a rationale for target-focused drug development or repurposing in DKD. Together, these data support a precision medicine approach to DKD, advancing the goal of early risk detection and individualized intervention based on genetic architecture and functional annotation.

Despite these promising findings, our study has several limitations. All analyses were conducted on individuals of Finnish ancestry, which may limit the generalizability of the results to other populations. Given the well-established genetic and environmental heterogeneity of DKD, future studies should prioritize replication in multi-ethnic cohorts. This will require population-specific eQTL reference panels, ancestry-matched GWAS summary statistics, and cross-tissue prediction models that are transferable across diverse backgrounds. The summary-level data used in this study do not include sex-stratified results, limiting our ability to examine sex-specific genetic effects. As DKD presents sex-dependent clinical and molecular features, future analyses incorporating sex-stratified or individual-level data may uncover associations that are masked in aggregated datasets and improve the biological relevance and precision of gene prioritization. Moreover, while our integrative framework provides strong statistical evidence for gene-level associations, the causal mechanisms linking these genes to DKD pathogenesis remain to be established. Functional validation in disease-relevant tissues and model systems will be essential to the determination of biological relevance and therapeutic tractability. Similarly, further functional validation experiments and multi-instrument MR analyses are essential for verifying the observed associations, which helps refine potential false-positive signals generated from large-scale TWAS screening and single-SNP analyses, particularly when interpreting complex bidirectional genetic effects. This is especially critical for interpreting bidirectional signals, which may arise from statistical artifacts when relying on single-SNP instruments. Together, these limitations highlight the need to extend transcriptomic mapping and gene prioritization efforts beyond single-ancestry cohorts and to complement association-based findings with mechanistic evidence to enable meaningful clinical translation.

In summary, our comprehensive cross-tissue TWAS and MR analysis has identified 13 susceptibility genes associated with DKD, of which four encode proteins with druggable targets. This significantly enhances our understanding of the genetic landscape of DKD. These discoveries offer valuable insights into the disease’s pathophysiology and open up avenues for therapeutic strategies, particularly in modulating immune response pathways, steroid hormone synthesis, DNA damage response (DDR), angiogenesis, and transcriptional regulation. Our findings establish a robust foundation for future functional investigations and therapeutic advancements in DKD.

## 5. Conclusions

In summary, our study represents the first systematic cross-tissue TWAS for DKD, coupled with rigorous MR validation, significantly advancing the field beyond traditional GWAS approaches. We have identified 13 susceptibility genes associated with DKD, with notably variable effect sizes. Preliminary but noteworthy signals were observed for genes with strong protective associations (*PRRT1*, *ZKSCAN8*) and pronounced risk associations (*VWA7*). Some of the identified genes exhibit pharmacological potential based on preliminary evidence or ongoing clinical development. These discoveries contribute meaningfully to the genetic characterization of DKD, offering new hypotheses about its molecular basis that should be further investigated in future functional and population-based studies. Our cross-tissue approach uniquely reveals tissue-specific effects that would remain hidden in conventional genetic studies, offering a valuable starting point for future functional validation and the exploration of potential therapeutic targets in DKD.

## Figures and Tables

**Table 1:** The druggability analysis of MR-validated genes.

Gene	Gene_id	Protein Names	Druggability Tier	Target Type	Drugs
*CYP21A2*	ENSG00000231852	Steroid 21-hydroxylase	3B	Clinical trial	BBP-631
*HLA-DRB1*	ENSG00000196126	HLA class II histocompatibility antigen, DRB1 beta chain	1	Successful	Glatiramer acetate; Apolizumab
*HLA-DRB5*	ENSG00000198502	HLA class II histocompatibility antigen, DR beta 5 chain	1	Clinical trial	Apolizumab
*MICB*	ENSG00000204516	MHC class I polypeptide-related sequence B	/	/	/
*MSH5*	ENSG00000204410	MutS protein homolog 5	/	/	/
*NOTCH4*	ENSG00000204301	Neurogenic locus notch homolog protein 4	3A	Clinical trial	Parsatuzumab (MEGF0444A); Crenigacestat (LY3039478)
*PHTF1*	ENSG00000116793	Protein PHTF1	/	/	/
*POU5F1*	ENSG00000204531	POU domain, class 5, transcription factor 1	/	/	/
*PRRT1*	ENSG00000204314	Proline-rich transmembrane protein 1	/	/	/
*VWA7*	ENSG00000204396	von Willebrand factor A domain-containing protein 7	/	/	/
*ZKSCAN8*	ENSG00000198315	Zinc finger protein with KRAB and SCAN domains 8	/	/	/
*ZNF165*	ENSG00000197279	Zinc finger protein 165	/	/	/
*ZSCAN9*	ENSG00000137185	Zinc finger and SCAN domain-containing protein 9	/	/	/

Note: Abbreviations: MR (Mendelian Randomization), HLA (Human Leukocyte Antigen), MHC (Major Histocompatibility Complex), *NOTCH* (Neurogenic locus notch homolog), *PHTF1* (Putative Homeodomain Transcription Factor 1), *POU5F1* (POU domain, class 5, transcription factor 1), *PRRT1* (Proline-rich transmembrane protein 1), *VWA7* (von Willebrand factor A domain-containing protein 7), KRAB (Krüppel-associated box), SCAN (SCAN domain), *MSH5* (MutS protein homolog 5), and *CYP21A2* (Cytochrome P450 21A2).

## Data Availability

Original data generated and analyzed during this study are included in the published article or the data repositories listed in the acknowledgments.

## Supplementary Materials

The following supporting information can be downloaded at: https://www.mdpi.com/article/10.3390/biomedicines13051231/s1, Supplementary Table S1. Candidate genes associated with DKD identified through sCCA; Supplementary Table S2. sCCA results for cross-tissue gene expression patterns in diabetic kidney disease; Supplementary Table S3. FUSION identified 346 significant genes associated with DKD; Supplementary Table S4. FUSION identified 100 genes that were significant in the cross-tissue sCCA analysis; Supplementary Table S5. COJO analysis of TWAS signals for diabetic kidney disease; Supplementary Table S6. MAGMA gene-based test identified 202 significant genes associated with DKD; Supplementary Table S7. The results of batch SMR analysis; Supplementary Table S8. FOCUS fine-mapping results of gene-trait associations; Supplementary Table S9. Mean and median FDR, PIP, and Z-scores of candidate genes identified by FOCUS fine-mapping (PIP > 0.8); Supplementary Table S10. Multi-tissue Mendelian randomization analysis results using GTEx V8 data; Supplementary Table S11. The functional pathway analysis of GeneMANIA related to 13 positive genes. Supplementary Figure S1. Heatmap of FUSION −logFDR values for DKD-associated genes across tissues; Supplementary Figure S2. ORA analysis of DKD-associated genes; Supplementary Figure S3. Tissue-specific expression of DKD-associated druggable genes.
