# Supplementary material for: A Cross-Tissue Transcriptome-Wide Association Study Reveals Novel Susceptibility Genes for Diabetic Kidney Disease in the FinnGen Cohort
Source: Biomedicines. 2025 May 19;13(5):1231. doi: 10.3390/biomedicines13051231 (PMC12108887; doi:10.3390/biomedicines13051231)
Supplement: Supplementary file 1 [file biomedicines-13-01231-s001.zip › Supplementary Figure S1.pdf]

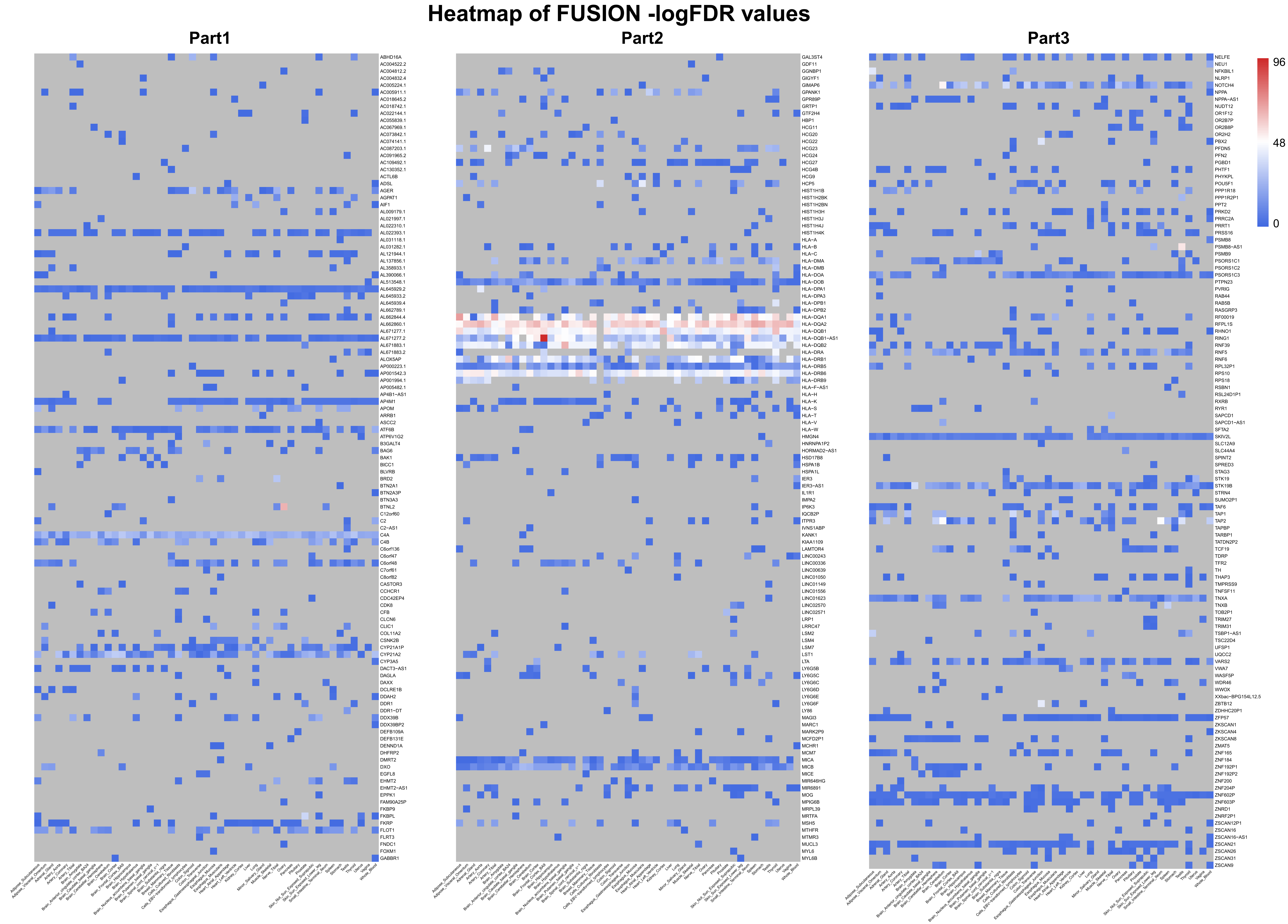

Supplementary Figure S1. Heatmap of FUSION -logFDR values for DKD-associated genes across tissues. This visualization shows 346 genes with significant associations (FDR<0.05) in at least one tissue. Higher values (red) indicate stronger statistical significance. Data presented in three panels for clarity.
