# Supplementary material for: A Cross-Tissue Transcriptome-Wide Association Study Reveals Novel Susceptibility Genes for Diabetic Kidney Disease in the FinnGen Cohort
Source: Biomedicines. 2025 May 19;13(5):1231. doi: 10.3390/biomedicines13051231 (PMC12108887; doi:10.3390/biomedicines13051231)
Supplement: Supplementary file 1 [file biomedicines-13-01231-s001.zip › Supplementary Figure S2.pdf]

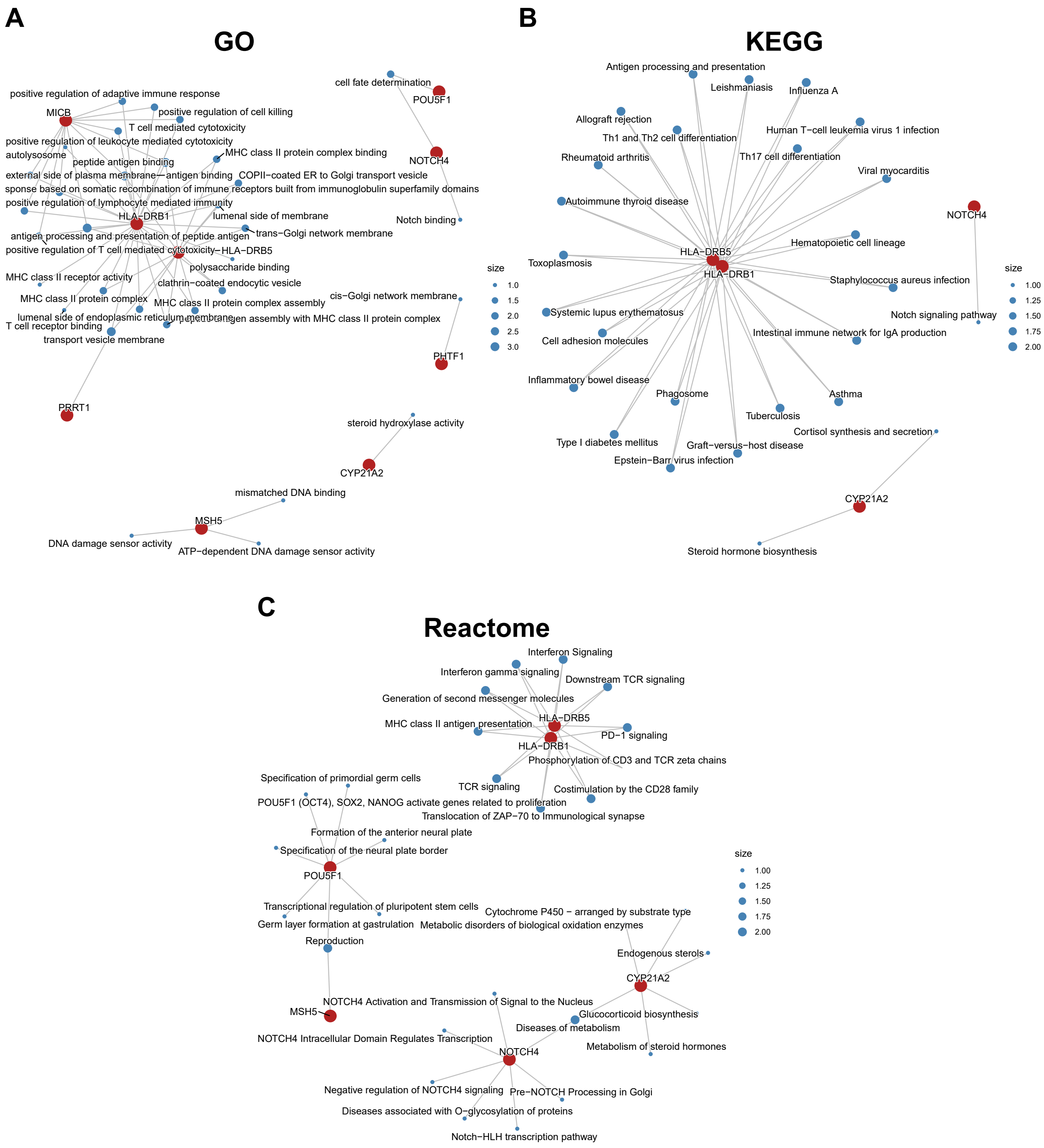

Supplementary Figure S2. ORA analysis of DKD-associated genes. Network visualization of ORA of functional enrichment indicated by GO (A), pathway enrichment indicated by KEGG (B), and Reactome (C) among identified genes. In the ORA plots, a red node indicates a positive gene in the enrichment analysis, and a blue node represents a positive term.
