# Supplementary material for: A Cross-Tissue Transcriptome-Wide Association Study Reveals Novel Susceptibility Genes for Diabetic Kidney Disease in the FinnGen Cohort
Source: Biomedicines. 2025 May 19;13(5):1231. doi: 10.3390/biomedicines13051231 (PMC12108887; doi:10.3390/biomedicines13051231)
Supplement: Supplementary file 1 [file biomedicines-13-01231-s001.zip › Supplementary Figure S3.pdf]

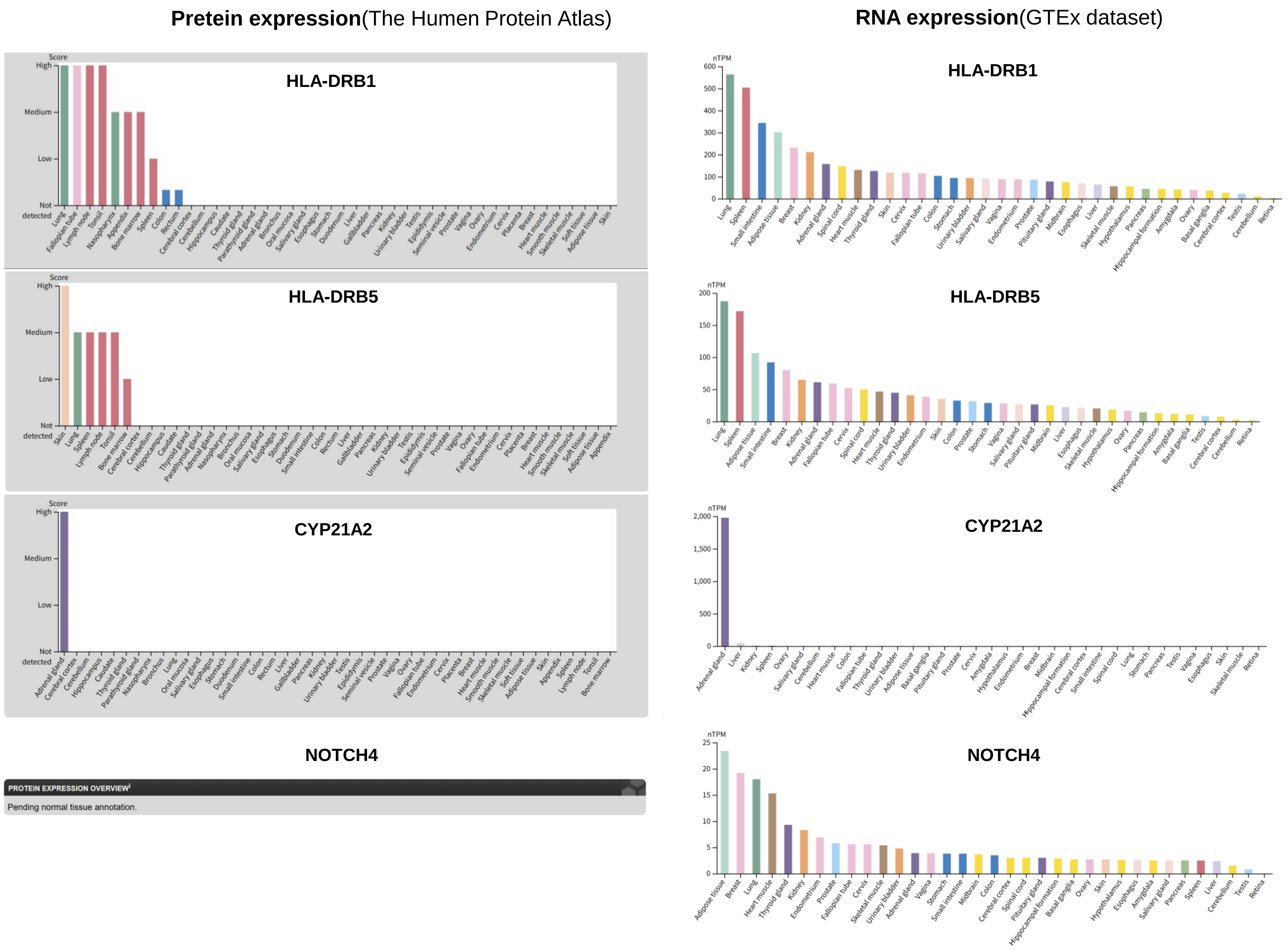

Supplementary Figure S3. Tissue-specific expression of DKD-associated druggable genes. Protein (left) and RN A(right) expression profiles of *HLA-DRB1, HLA-DRB5, CYP21A2*, and *NOTCH4* across human tissues, showing tissue-specific expression patterns relevant to DKD pathophysiology. All images are from The Human Protein Atlas website (https://www.proteinatlas.org/).
